# 

**Published:** 1891-11

**Authors:** 


CONTENTS:

PAGE

ORIGINAL ARTICLES:

Barren Mares. By C. C. Lyford, M. D., D. V. S. .... 587
Fistulous Opening from the Mouth. By Daniel D. Lee, M. D., V... 606
Measles in Horses. By same .... 609
Identification of Animals. By R. S. Huidekoper, M. D., Vet. .... 610
Abscess of Gutteral Pouch. By W. H. Ridge, V., M. D. .... 620
Age of Sheep and Goat. By R. S. Huidekoper, M. D., Vet. .... 623
Recent Patents .... 631
Cases: By Herbert Neher .... 634
By Thomas B. Rogers .... 635
Iowa State Veterinary Medical Association .... 637
The Illinois State Veterinary Medical Association .... 637

SELECTIONS FROM FOREIGN JOURNALS:

German Review .... 638
English Review .... 641

COMMUNICATIONS; .... 642

SOCIETY PROCEEDINGS:

Pennsylvania State Veterinary Medical Association .... 646
Keystone Veterinary Medical Association .... 650
Kansas Veterinary Medical Association .... 653
South Dakota Veterinary Medical Association .... 654
The Philadelphia Society of Veterinary Medicine .... 654

REVIEW DEPARTMENT:

The Comparative Anatomy of Domesticated Animals .... 655
U. S. Department of Agriculture .... 655
Epidemic Influenza .... 656
Books and Pamphlets Received .... 656

